# Overdrive pacing of spiral waves in a model of human ventricular tissue

**DOI:** 10.1038/s41598-020-77314-5

**Published:** 2020-11-26

**Authors:** Sergei F. Pravdin, Timofei I. Epanchintsev, Alexander V. Panfilov

**Affiliations:** 1 Krasovskii Institute of Mathematics and Mechanics, Sector of Mathematical Modelling in Cardiology, Yekaterinburg, Russia 620108; 2 HPC Department, Ural Federal University, Yekaterinburg, Russia 620083; 3 Biomed Lab, Ural Federal University, Yekaterinburg, Russia 620083; 4 Faculty of Sceince, Ghent University, 9000 Ghent, Belgium; 5 Arrhythmia Department, Almazov National Medical Research Centre, Saint Petersburg, Russia 197341

**Keywords:** Computational biophysics, Cardiac device therapy, Arrhythmias, Computational science, Biological physics

## Abstract

High-voltage electrical defibrillation remains the only reliable method of quickly controlling life-threatening cardiac arrhythmias. This paper is devoted to studying an alternative approach, low-voltage cardioversion (LVC), which is based on ideas from non-linear dynamics and aims to remove sources of cardiac arrhythmias by applying high-frequency stimulation to cardiac tissue. We perform a detailed in-silico study of the elimination of arrhythmias caused by rotating spiral waves in a TP06 model of human cardiac tissue. We consider three parameter sets with slopes of the APD restitution curve of 0.7, 1.1 and 1.4, and we study LVC at the baseline and under the blocking of INa and ICaL and under the application of the drugs verapamil and amiodarone. We show that pacing can remove spiral waves; however, its efficiency can be substantially reduced by dynamic instabilities. We classify these instabilities and show that the blocking of INa and the application of amiodarone increase the efficiency of the method, while the blocking of ICaL and the application of verapamil decrease the efficiency. We discuss the mechanisms and the possible clinical applications resulting from our study.

## Introduction

Sudden cardiac arrest, which in most cases occurs as a result of arrhythmia called ventricular fibrillation (VF), is the most common cause of death in industrialized countries^1^. Another type of arrhythmia, atrial fibrillation (AF), is the most common sustained arrhythmia in clinical practice and is a major risk factor for stroke^2^. There are also many other types of heart rhythm disturbances, called tachyarrhythmias, which are associated with less complex electrical activity of the heart.

An effective way to stop these types of arrhythmias is electrotherapy, whereby one or a series of electric impulses are applied via electrodes. The goal is to return the abnormal heartbeat to a normal rhythm. The electrotherapy methods can be classified by the electrode placement and by the stimulation energy. The electrodes can be placed on the body of a patient or directly on the heart (e.g. during surgical intervention). In the latter case, they can be stimulated by a small battery-equipped device implanted under the patient’s skin. The implanted device is closest to the myocardial cells and uses the lowest energy to excite the muscle.

In general, the impulses can be divided into three energy classes, the highest-energy *shocks*, which use voltages about 200 V or more, *low-voltage shocks*, which use potentials between 10 and 100 V, and *pacing stimuli*, which use less than 10 V but usually more than 1 V.

Biophysical mechanisms of those treatments have been studied for about a century. The most widely used methods are the shock electrotherapy, which is called *defibrillation* in the case of VF, and *cardioversion* in other cases. The idea of the shock electrotherapy is to depolarize all heart cells using one stimulus and reset arrhythmic excitation pattern to normal cardiac excitation. However, such a shock results in serious adverse effects^3^. Reducing the required amount of energy, for example by decreasing the voltage of an electric shock, is a classical problem in biomedical research. Over the years, various ways to do that as well as to increase efficacy of the electrotherapy and to decrease complication risks have been proposed. For example, multi-stage electrotherapy^4^, which involves a sequence of three series of stimuli, first shocks, second low-voltage shocks and finally pacing, and low-energy anti-fibrillation pacing^5^, which is a series of low-voltage shocks, have been tested in experiments. Some prospective ideas are based on theoretical considerations, such as resonant drift^6,7^. Unfortunately, biophysical mechanisms of the low-voltage shock therapy are still poorly understood. It is largely unknown why this therapy succeeds or fails in specific cases.

Biophysics of the pacing treatment is based on staged interaction between pathological electrical excitation waves and waves which emerge from the electrode. Rotational electrical activity, including rotating spiral waves, is one of the important mechanisms of many dangerous cardiac arrhythmias. Superseding of a spiral wave by a train of plane waves is called *overdrive pacing* (ODP). There is another term, *anti-tachycardia pacing* (ATP). Some researchers say that ODP is a subtype of ATP^8^ while others, vice versa^9^.

The mechanisms of suppression of the spiral waves by ODP were studied in early papers by Krinsky and Agladze^10^, who performed experiments using chemical excitable media and who found that elimination occurs via induced drift of the spirals. The external wavetrain induced drift of the spiral wave and pushed its tip to the boundary of the medium where the spiral wave annihilated. This process was also studied numerically^11^ in a simplified low-dimensional model of cardiac tissue. In a more recent paper, Stamp et al.^12^ studied ODP using a Luo–Rudy I model^13^. They found that pacing with a period shorter than the period of the spiral can suppress the spiral wave activity, but the results were sometimes worse than expected. The mechanisms of unsuccessful ODP were not addressed. More recent studies have considered ODP for a spiral wave anchored to a heterogeneity such as a post-infarction scar. It was shown that successful elimination requires unpinning of the spiral wave as a first step. This can be done using far-field electric pacing^14^, a theoretically-studied method involving application of an electric field at a distance from the heart rather then passing an electric current.

The ODP has been widely used in clinical practice and studied theoretically. The rapid electrical pacing can terminate atrial flutter^15^ and ventricular tachycardia (VT)^16^. Currently, ODP/ATP is a part of implantable cardioverter-defibrillator programs and has good efficiency of 78–86% in cases of VT^17,18^ and 41–81% in cases of fast VT^19^. However, the optimal pacing parameters and mechanisms of therapy failures remain unclear. In addition, this methodology does not work for AF or VF and can even induce fibrillation as a side effect. Therefore, it is important to study the fundamental mechanisms behind the ODP that can stop arrhythmia.

We will call this method *low-voltage cardioversion* (LVC) to emphasize that we study cardioversion via the mechanism of interaction of arrhythmia waves with waves produced by low-amplitude stimuli. We studied LVC in silico in an Aliev–Panfilov model^20^ using isotropic^21,22^ and anisotropic media^23^ and in an LR-I model using anisotropic media^24,25^. We found that stimulation period, electrode location and tissue anisotropy affect the success of the procedure. We also performed a few simulations^25–27^ using a TP06 model of human ventricular tissue^28^. We showed that the elimination of spiral waves in the TP06 model is much more challenging and occurs in a much narrower range of periods compared to low-dimensional models and the LR-I model. This highlights the importance of studying LVC in more realistic models of human cardiac tissue. However, the results^25^ for the TP06 model are rather preliminary; tests were performed only for one set of parameters, and it was not clear how the properties of cardiac tissue can affect LVC. The possible mechanisms of LVC improvement were not studied. In addition, the pacing by itself can result in dynamic instabilities that strongly depend on the parameters. As the elimination of spiral waves in myocardium not affected by drugs is extremely difficult, it would be interesting to study whether the blocking of major ionic currents and the application of anti-arrhythmic drugs may increase the efficiency of LVC.

The aim of this paper is to perform extensive numeric studies of LVC in a model of human ventricular tissue. We study how changes in the major ion currents (INa, ICaL) and the application of the drugs amiodarone and verapamil affect the success of LVC. We also study three parameter sets describing cardiac tissue with different degrees of dynamic instabilities controlled by the slope of the action potential duration (APD) restitution curve and discuss the mechanisms of the observed effects.

## Methods

### Mathematical model

We used the monodomain reaction–diffusion system^29^ in the form

$$\begin{aligned} \frac{\partial u}{\partial t}& = D \Delta u -\frac{I_{\mathrm{ion}}+ I_{\mathrm{stim}}}{C_m},\\ I_{\mathrm{ion}}& = I_{Na}+I_{to}+I_{Kr}+I_{K1}+I_{NaCa}+I_{NaK}+I_{pCa}+I_{pK}+I_{bNa}+I_{bCa}+I_{CaL}+I_{Ks}, \end{aligned}$$

where $u=u(\vec{r},t)$ is the cell transmembrane potential at points $\vec{r}=(x,y)$ at time *t*, $0\le x,y\le L$, *D* is the diffusion coefficient, $\Delta u=u_{xx}+u_{yy}$ is Laplacian in two dimensions, $I_{\mathrm{stim}}=I_{\mathrm{stim}}(\vec{r},t)$ is the external stimulation current and $C_m$ is the cell membrane capacitance. For ionic currents, we used the TP06 model^28^ and considered three parameter sets with slopes of the APD restitution curve of 0.7, 1.1 and 1.4 listed in Table 1.

**Table 1:** Modifications of the TP06 model with different slopes of the APD restitution curve (from [^28^, Table 2]).

Slope	$G_{Kr}$	$G_{Ks}$	$G_{pCa}$	$G_{pK}$	$\tau _f$ inact	Notation
0.7	0.134	0.270	0.0619	0.0730	$\times 0.6$	SL07
1.1	0.153	0.392	0.1238	0.0146	Normal	SL11
1.4	0.172	0.441	0.3714	0.0073	$\times 1.5$	SL14

### Drug effects

The effect of amiodarone was modelled in a recent work^30^ using the same cell-level model TP06 and the Hill rule. Six ionic currents were changed by blocking factor *c* (different for each current) depending on the drug concentration [*D*], the half maximal inhibitory concentration $IC_{50}$ and the Hill coefficient *H* in the following way:

$$\begin{aligned} c = \frac{[D]^{H}}{[D]^{H}+IC_{50}^{H}}. \end{aligned}$$

Note that the two last parameters, $IC_{50}$ and *H*, are different for each ionic current. We followed this approach and examined two amiodarone concentrations, 1 μM as a low concentration and 3 μM as a high concentration. The affected ionic currents are listed in Table 2; the blocking factor values were taken from [^30^, Table 2].

The effects of verapamil were modelled by blocking IKr and ICaL. These currents were chosen because it was shown [^31^, Figs. 9 and 10] that verapamil mostly affects IKr and ICaL and does not affect late INa, peak INa, Ito, IKs or IK1. All modifications of the parameters are given in Table 2.

**Table 2:** Modifications of the TP06 model with changed ionic currents.

Drug	Model parameter	Multiplicator name (if used) and value	Notation
Class I drug	GNa	c_GNa=0.75	Na75
Class I drug	GNa	c_GNa=0.5	Na50
Class I drug	GNa	c_GNa=0.25	Na25
Verapamil	GCaL	0.75	ver75
	GKr	0.75	
Verapamil	GCaL	0.50	ver50
	GKr	0.50	
Verapamil	GCaL	0.25	ver25
	GKr	0.25	
Class IV drug	GCaL	0.75	CaL75
Class IV drug	GCaL	0.50	CaL50
Class IV drug	GCaL	0.25	CaL25
Amiodarone low concentr. ($1\,\upmu \mathrm{M}$)	GKr	0.7128	amio1
	GNa	0.7633	
	ipmax	0.9398	
	GCaL	0.8529	
	knaca	0.7674	
	GKs	0.6942	
Amiodarone high concentr. ($3\,\upmu \mathrm{M}$)	GKr	0.4841	amio3
	GNa	0.5878	
	ipmax	0.8387	
	GCaL	0.6591	
	knaca	0.5238	
	GKs	0.5373	

### Pacing protocols

We used an S1–S2 cross-field protocol to generate spiral waves. We implemented these two stimuli S1 and S2 by setting the voltage to $+50\,\mathrm{mV}$. The first stimulus was applied to a narrow strip $x\le 10\,\mathrm{mm}$ at the left side of the square domain so that a plain wave propagated toward the right square edge. The second stimulus was applied at time $t_{S2}$ to a rectangle $0\le x\le L$, $\alpha _{S2}L\le y\le L$ so that the first wave back front was intersected and a spiral wave emerged and rotated approximately at the domain centre. Parameters $t_{S2}$ and $\alpha _{S2}$ are given in Supplementary Tables S1–S3.

The ODP was applied by using a current. The stimulation current was equal to $I_{\mathrm{st}}$ and was applied to region $\Omega _{\mathrm{stim}}$ with period $T_{\mathrm{stim}}$ by pulses, with duration $t_{\mathrm{stim}}$ starting from $\tau _0$:

$$\begin{aligned} I_{\mathrm{stim}}(x,y,t) = {\left\{ \begin{array}{ll} I_{\mathrm{st}}, \text { if }(x,y)\in \Omega _{\mathrm{stim}},\ t\ge \tau _0,\ \left\{ \frac{t-\tau _0}{T_{\mathrm{stim}}}\right\} \le \frac{t_{\mathrm{stim}}}{T_{\mathrm{stim}}};\\ 0, \ \ \ \text { otherwise.} \end{array}\right. } \end{aligned}$$

The stimulation was started after the spiral wave occupied the entire computational domain.

The common computation parameters are given in Table 3. The stimulation was applied from one long linear electrode $\Omega _{\mathrm{stim}}: 0\le x\le 2.4\,\mathrm{mm}$ occupying the left part of the domain. The stimulation began at moment $\tau _0$ depending on the model parameters (see Supplementary Tables S1–S3).

Our previous simulations^25,26^ showed that pacing at a constant rate often led to the appearance of additional spiral waves at the electrode. Additional spiral waves can occur via a mechanism similar to the one used in the S1S2 protocol when an electrode is stimulated at the vulnerable phase of the action potential. Such additional spiral waves can either make LVC impossible or in some cases can help LVC, as new spirals can annihilate with the original one.

In this paper, we focus on the process of eliminating spiral waves due to interaction with a faster excitation source without taking into account possible new spirals which can occur due to stimulation at the vulnerable phase, as this undesirable phenomenon is not directly related to the stimulation period and can occur at any pacing rate. To that end, we implemented the following procedure. To begin, we found conditions (an initial pacing period and a number of pulses) when the wavetrain captured an area around the electrode. We then saved the state variables and continued the periodic pacing with the target period. If the target period was less than the initial period, we decreased the pacing period by 1 ms until we reached the target one. This procedure prevented stimulation of the electrode at the vulnerable phase and the onset of new spiral waves. The results for the constant stimulation period for a few of the parameter values can be found in Supplementary Data Section B.

### Numeric and computer implementation

We used the explicit Euler method for the integration of the system. The grid was uniform. Laplacian was computed using the five-point stencil.

Spiral wave dynamics were assessed by tracing the spiral wave tip $\vec{r} _{tip}(t)$^29^ using the method described in^32^. Thus, we solved the system of the two algebraic equations

$$\begin{aligned} u( \vec{r} _{tip},t)=u^*, \qquad u( \vec{r} _{tip},t+\Delta t)=u^*, \end{aligned}$$

where $u^*=0\,\mathrm{mV}$ and $\Delta t=10\,\mathrm{ms}$ are parameters of the method; *u* is the potential given on the grid *x*, *y*, *t*. The solution of the system is $\vec{r} _{tip}(t+\Delta t)$. If the system had several solutions, we concluded that there were several spiral waves.

The spiral wave period was assessed by tip tracing in cases without meandering. We measured the duration of 20 rotations of the tip. In cases with meandering, we measured the duration of 20 action potentials, including diastolic intervals, at a point far from the tip and the domain boundary.

**Table 3:** Mesh, stimulation and diffusion parameters.

Parameter	Value
Spatial grid size, mm	0.25
Time step, ms	0.02
Stimulation duration $t_{\mathrm{stim}}$, ms	1.5
Diffusion coefficient *D*, $\mathrm{mm}^2/\mathrm{ms}$	0.154
Integration domain size *L*, mm	100

The numerical solver was implemented with the C programming language, using the OpenMP library for parallel execution of the code. Visualization of results was done using the Python programming language and matplotlib. Computations were performed with double precision and run on URAN cluster (IMM UB RAS; URAN has Intel(R) Xeon(R) CPU E5-2697 v4, X5675, E5-2660, E5-2650, E5450 machines).

## Results

### Results of the LVC at baseline

**Figure 1:**
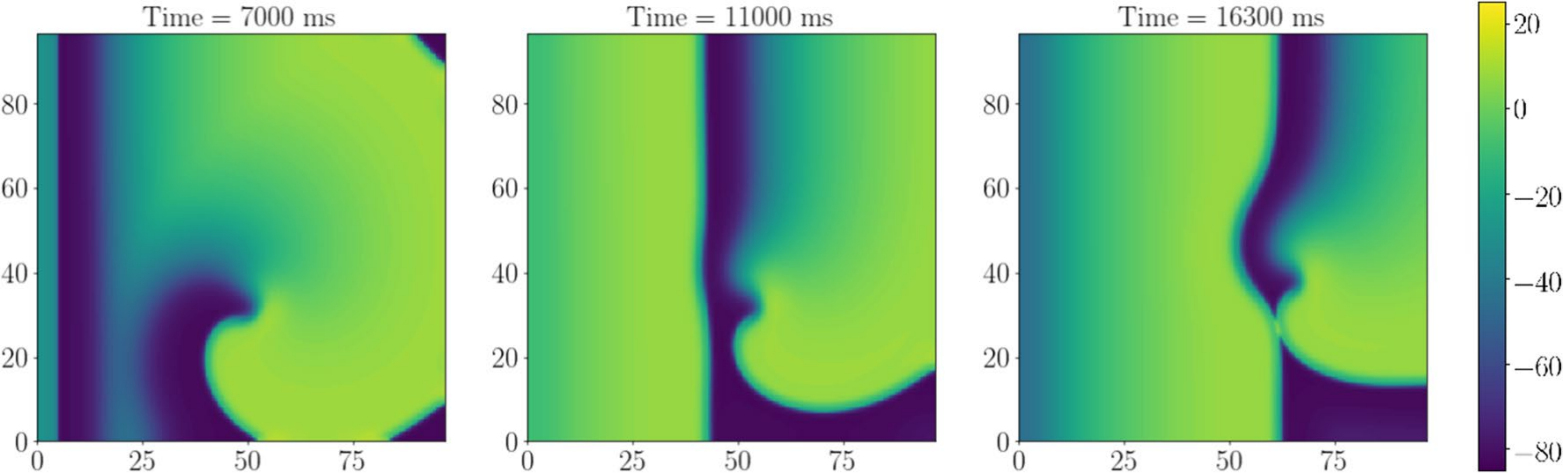
Example of successful LVC with a period shorter than that of the spiral wave. X-axis, mm, is horizontal; Y-axis, mm, is vertical; colour shows potential, mV (colour legend is at the right). SL07, $T_{\mathrm{stim}}=228\,\mathrm{ms}$, $T_{\mathrm{sw}}=237.6\,\mathrm{ms}$, relative pacing period is 0.96. See animation in Supplementary video 1.

Figure 1 shows an example of the LVC for parameter set SL07, which corresponds to an APD restitution curve with minimal slope 0.7. We see that the application of external waves with a period $T_{\mathrm{stim}}$ shorter than the spiral wave period $T_{\mathrm{sw}}$ progressively shifted the spiral to the right boundary of the tissue where it finally disappeared. A similar situation (not shown) occurred for parameter set SL11 (corresponding to the greater slope of the APD restitution curve 1.1).

**Figure 2:**
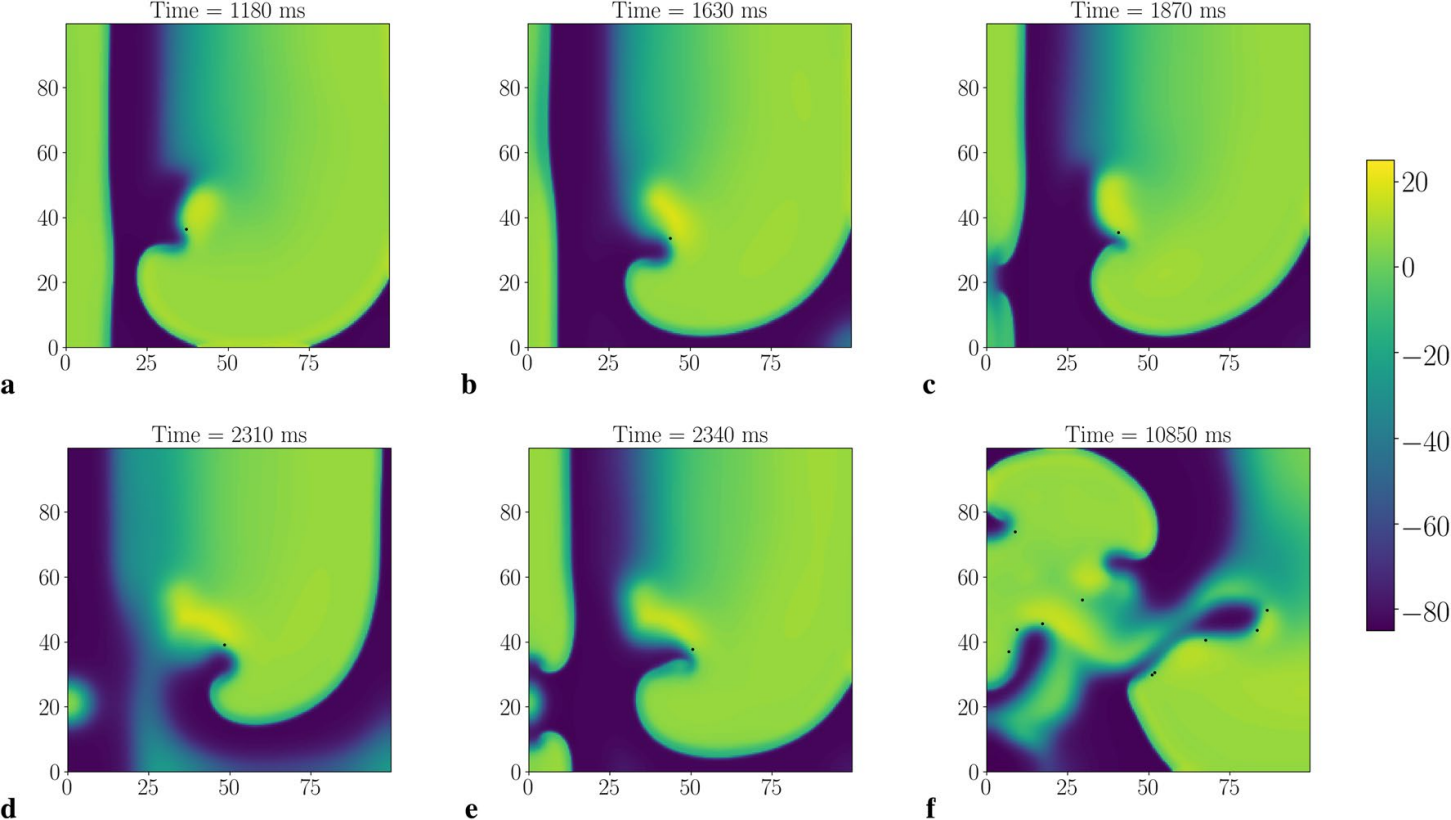
Example of unsuccessful LVC with the ‘Conduction block’ dynamic instability. X-axis, mm, is horizontal; Y-axis, mm, is vertical; colour shows potential, mV (colour legend is at the right). The tip of the spiral wave is shown as a black dot. SL14, norm, $T_{\mathrm{stim}}=232\,\mathrm{ms}$. We see the acceleration of a plain wave at the bottom left part of the tissue (**b**), then the tissue is not ready for the next stimulus (**c**), long action potential hinders the stimulation (**d**). See animation in Supplementary video 2.

However, stimulation for the parameter set SL14 (corresponding to the maximal slope of the APD restitution curve 1.4) resulted in a different outcome. The stimulation either did not reach the spiral (if $T_{\mathrm{stim}}>T_{\mathrm{sw}}$, not shown) or resulted in the onset of complex spatio-temporal patterns of various types. Figure 2 shows an example of such a pattern at several time moments for stimulation period $T_{\mathrm{stim}}=232\,\mathrm{ms}$ (period of spiral wave $T_{\mathrm{sw}}=240\,\mathrm{ms}$). We see that the wave train was not able to reach and push the original spiral away, and so the LVC was impossible.

Such complex dynamics are a result of dynamic instabilities that occur in tissue under high-frequency pacing. Instability at the stimulating electrode did not allow us to obtain a wavetrain with the period faster than the period of spiral, as alternations in APD eventually produced a 2:1 Wenckebach block^33^. The interaction of the waves from the electrode with the original spiral wave produced a sustained complex spatio-temporal pattern that is depicted in Fig. 2. We further illustrate that additional wave breaks are the result of high-frequency pacing in supplementary Fig. S1. There we show that the number of additional wave breaks (phase singularities) was always smaller in the absence of external pacing in comparison with the paced tissue by up to 5–8 tips.

Because this pattern was a result of two processes—instabilities that occurred at the electrode and the interaction of high-frequency waves with the original spiral—we first quantified the electrode instabilities and then considered their interaction with the spiral.

### Dynamic instabilities at high-frequency pacing

**Figure 3:**
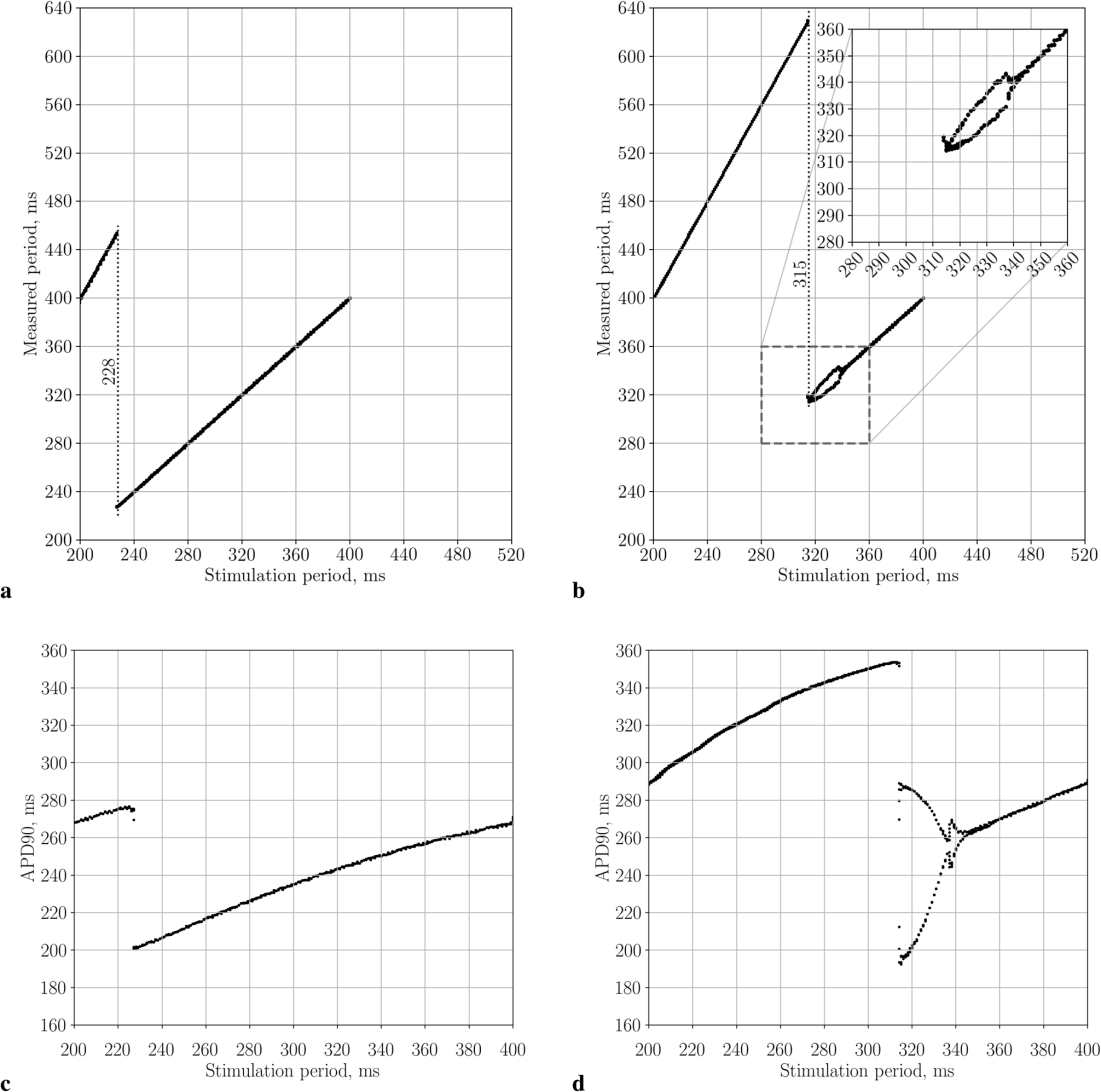
Measured wave periods in 1D cable depending on the stimulation period. Models with parameters sets SL07 (**a**) and SL14 (**b**). APD-90 in SL07 model (**c**) and SL14 model (**d**). Dotted lines indicate $T_{\mathrm{1D}}$.

We studied the manifestation of the dynamic instabilities that can occur as a result of high-frequency pacing for the parameter values used in our 2D simulations in absence of the spiral wave. Figure 3 shows the results of high-frequency pacing with different periods for the parameter sets SL07 and SL14. For set SL07 (Fig. 3a), we see that the period measured at 47 mm from the stimulation electrode was equal to the stimulation period for $T_{\mathrm{stim}}> 227\,\mathrm{ms}$ and a 2:1 conduction block occurred for $200< T_{\mathrm{stim}}< 227\,\mathrm{ms}$. For parameter set SL14, the measured periods were equal to the stimulation periods for $T_{\mathrm{stim}}> 344\,\mathrm{ms}$ and a 2:1 conduction block occurred for $200< T_{\mathrm{stim}}< 314\,\mathrm{ms}$. However, the pacing with $314 \le T_{\mathrm{stim}}\le 344\,\mathrm{ms}$ showed alterations in period values. This is typical for the alternans instability that occurs at a steep slope of the APD restitution curve^34,35^. Its manifestation is more clear in Fig. 3d, where we show APD90 for the same simulation. We see huge variation in the APD values during instability. Because the instability amplitude increased with the decrease in the stimulation period, one of the stimulated waves eventually bumped into the longer tail of the preceding wave and disappeared. As a result of this instability, we saw the onset of a 2:1 conduction block for $T_{\mathrm{stim}}<314\,\mathrm{ms}$. Note that the onset of the conduction block for parameter set SL07 is logical because it occurred when the stimulation period reached the refractory period of the action potential. However, for parameter set SL14, the 2:1 block occurred at much longer periods of $T_{\mathrm{stim}}<314\,\mathrm{ms}$. Therefore, we see that the tissue with parameter set SL14 could not be stimulated with the period faster than 314 ms. Consequently, we could not produce a wave train faster than the SW period and could not remove the spiral wave.

If we stimulated faster than the period of the spiral wave, for example, with a constant period $T_{\mathrm{stim}}=232\,\mathrm{ms}$, the real stimulation period would become $T_{\mathrm{stim}}=464\,\mathrm{ms}$ due to the 2:1 block (see Fig. 3b) and the waves from the rotating spiral could reach the electrode at some places and locally affect its excitation. As a result, we would get wave blocks at the electrode. We call this type of interaction, when wave blocks occur as a result of interaction between the stimuli and the original spiral wave, a conduction block (CB) type of instability. An example can be seen in Fig. 2. Waves from the electrode area interact with the original spiral wave and create the complex spatio-temporal excitation pattern shown in Fig. 2f that cannot be removed by LVC.

Overall, the studies of the instabilities shown in Fig. 3 can explain the results of LVC for parameter sets SL07, SL11 and SL14. Indeed, the period of the spiral wave for set SL07 is $T_{\mathrm{sw}}=237.6\,\mathrm{ms}$, and a 2:1 block occurred for $T_b=227\,\mathrm{ms}$; thus, minimal assimilable period in 1D is $T_{\mathrm{1D}}=228\,\mathrm{ms}$; $T_{\mathrm{1D}}<T_{\mathrm{sw}}$ and LVC was successful. For model SL11, $T_{\mathrm{1D}}=228\,\mathrm {ms}<T_{\mathrm{sw}}=240\,\mathrm{ms}$, and LVC was again successful. However, for set SL14, $T_{\mathrm{1D}}=315\,\mathrm{ms}$ while the SW period was $T_{\mathrm{sw}}=243\,\mathrm{ms}$, making $T_{\mathrm{1D}}>T_{\mathrm{sw}}$ and LVC unsuccessful.

Now let us apply such consideration to a wider range of parameters. In particular, we will study how the application of the drugs and the blocking of major ionic currents affect the LVC and whether its efficiency can be improved by the pharmacological actions.

### Effect of the drugs and the ionic current blocks on the LVC

Here, we study whether the blocking of ionic currents or the application of common anti-arrhythmic drugs can improve LVC effectiveness. We chose to examine the blocking of INa, which is the main effect of Class I anti-arrhythmic drugs, the blocking of ICaL, the application of verapamil (Class IV) and the application of amiodarone (Class III anti-arrhythmic drug).

#### Effect of INa block

**Figure 4:**
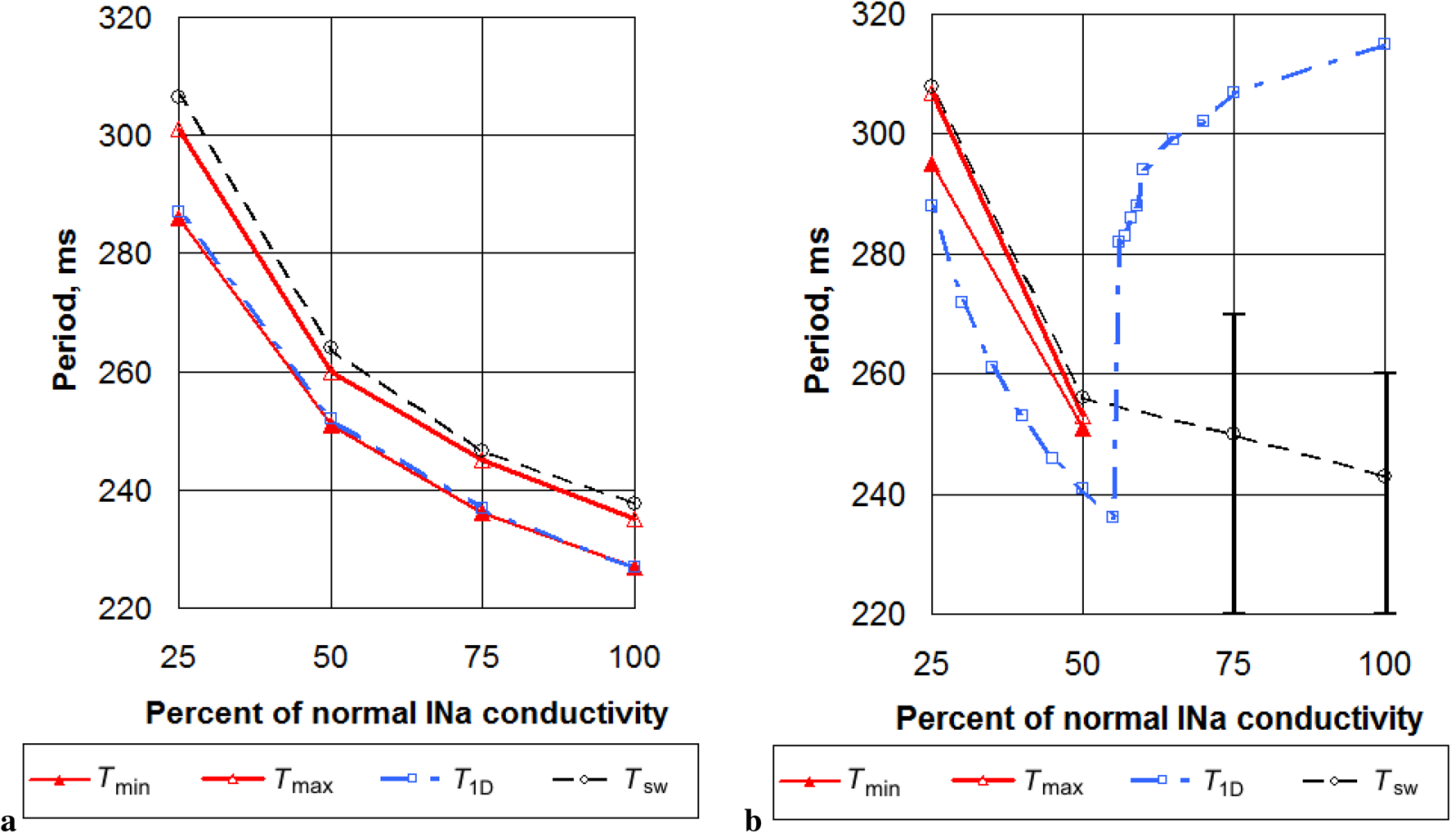
Limits of the effective absolute stimulation periods (red solid lines) in models with normal and decreased conductivities for Na ionic current. No limits are shown in cases of unsuccessful LVC. Slopes 0.7 (**a**), 1.4 (**b**). Spiral wave periods (black dashed lines) and their limits in cases of meandering (vertical black segments); minimal assimilable pacing periods (blue dotted-and-dashed lines).

 Our results for this case are shown in Fig. 4.

**Figure 5:**
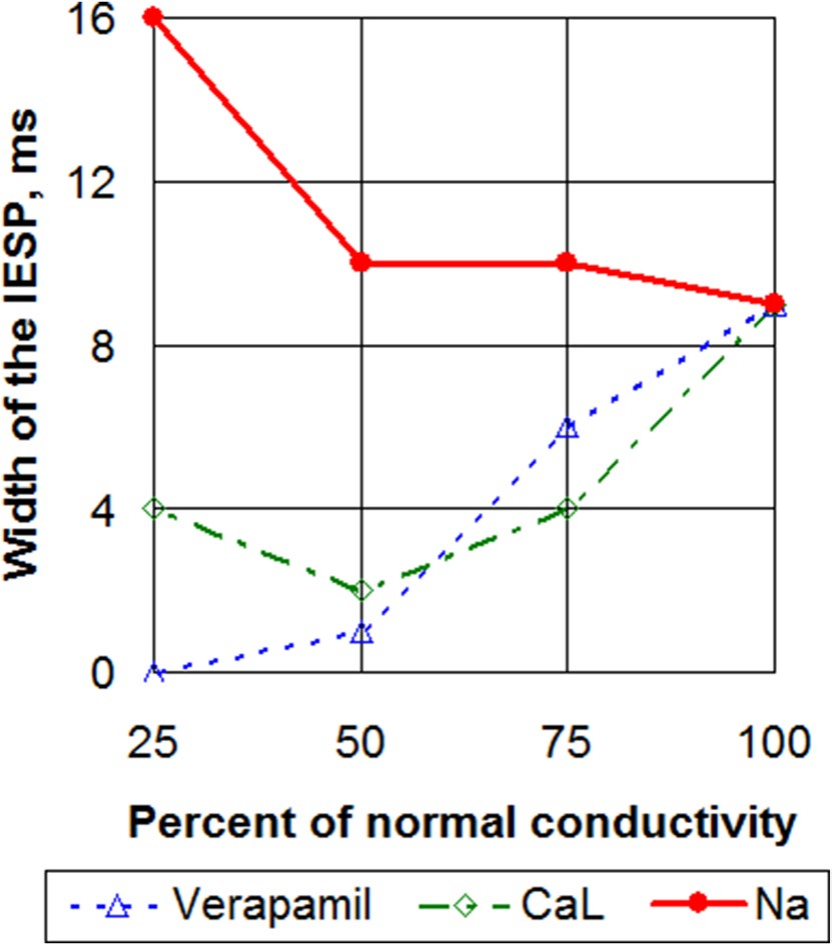
The width of the interval of effective stimulation periods for models with normal and decreased conductivities for CaL+Kr (verapamil effect), CaL and Na ionic currents. SL07 case.

For parameter set SL07, we see a slight increase in the efficiency of LVC with c_GNa decrease (see Fig. 5). Indeed, for Na75 the width of the possible LVC periods window was 10 ms, while for Na25 it was 16 ms. The effective LVC periods formed an area bounded by the minimal $T_{\min }$ and maximal $T_{\max }$ effective LVC periods (see Fig. 4a). We see that the minimal period $T_{\min }$ could be well approximated by the minimal assimilable pacing period $T_{\mathrm{1D}}$. The latter period $T_{\mathrm{1D}}$ was obtained from 1D simulations (see Fig. 3) as the minimal period at which no 2:1 block occurred. The period $T_{\max }$ was close to the period $T_{\mathrm{sw}}$. Both periods $T_{\min }$ and $T_{\max }$ increased with the decrease of c_GNa.

For parameter set SL14, the situation was different (see Fig. 4b). At the baseline, LVC was impossible, the spiral wave meandered, and its period in different points fluctuated between 220 ms and 260 ms. These spiral wave periods were less than $T_{\mathrm{1D}}=315\,\mathrm{ms}$. A similar pattern was observed in the case of Na75, in which the spiral wave period fluctuated between 220 ms and 270 ms, and $T_{\mathrm{1D}}$ was 307 ms. LVC was possible only in cases Na25 and Na50 in which no meandering was observed and the spiral wave period was uniform in all nodes situated far enough from the core and domain boundary.

**Figure 6:**
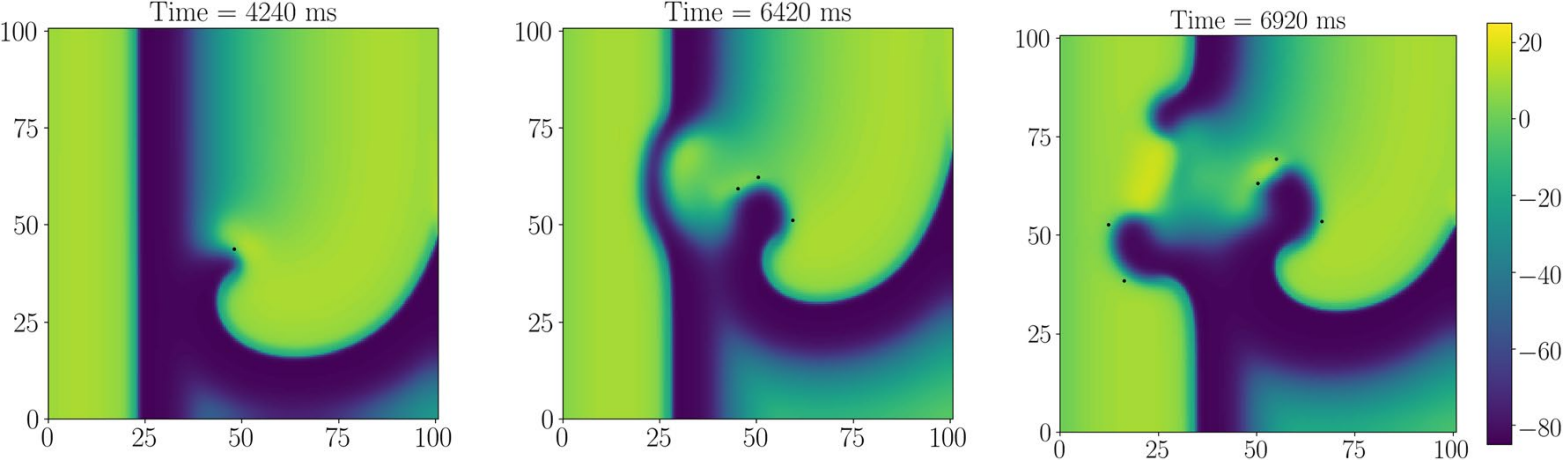
Dynamic instability of the core type. The spiral wave is stable without the stimulation (on the left). When the wave train starts to touch its core zone, the spiral wave breaks up (in the middle). The instability leads to a global break-up (on the right). X-axis, mm, is horizontal; Y-axis, mm, is vertical; colour shows potential, mV (colour legend is at the right). SL11; Na75; $T_{\mathrm{stim}}=239\,\mathrm{ms}$. See animation in Supplementary video 3.

**Figure 7:**
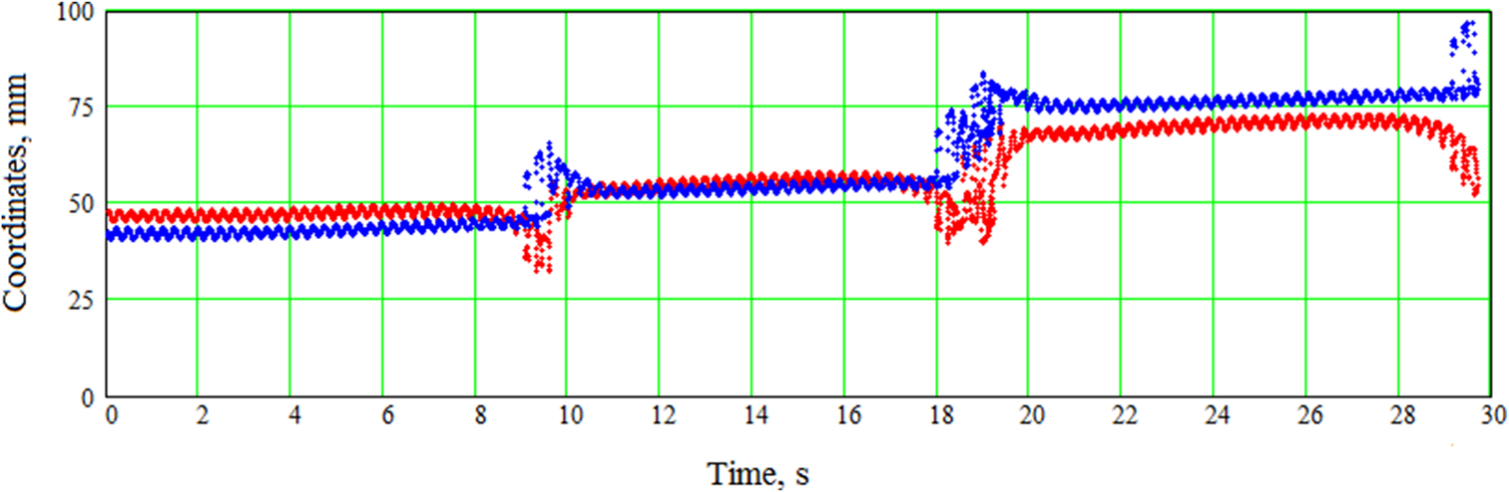
Example of transient core-type dynamic instability (jumps-type). Tip coordinates, mm, are plotted against time, s. The red line shows the *x* coordinate and the blue line shows the *y* coordinate. The spiral wave breaks up near its core as a result of the stimulation. The core then moves to another position and the wavefront annihilation zone shifts back toward the electrode. SL11; Na75; $T_{\mathrm{stim}}=247\,\mathrm{ms}$. See animation in Supplementary video 4.

The period $T_{\mathrm{1D}}$ non-monotonically depended on the c_GNa. When c_GNa decreased from 100% to 56%, the $T_{\mathrm{1D}}$ period decreased smoothly, but there was an abrupt drop between c_GNa=55% and 56%. A further decrease in c_GNa caused a monotonic increase in $T_{\mathrm{1D}}$. At the same time, the spiral wave period monotonically increased. For c_GNa $\le 50\%$, $T_{\mathrm{sw}}$ became longer than $T_{\mathrm{1D}}$, and therefore LVC was possible. In Fig. 4b, we see that $T_{\mathrm{sw}}$ approximated well the upper boundary $T_{\max }$ of the successful LVC, while the minimal effective stimulation period $T_{\min }$ was up to 10 ms longer than $T_{\mathrm{1D}}$. This difference is caused by dynamic instabilities. However, dynamic instabilities in this interval of periods resulted from the presence of a spiral wave. Figure 6 shows an example of such instability, which we call the ‘core’ type of instability. We see that the spiral wave interacted with the plane wave from the electrode and additional wavebreaks appeared at dynamic islands of elongated APD. Note that such instability does not necessarily lead to LVC failure. Figure 7 shows an example when a core-type instability was transient. The spiral wave broke at the core when it interacted with the plane wave. However, the additional spirals annihilated, and the original spiral persisted and moved to another place after the annihilation. The spiral wave was eventually removed. This kind of scenario usually occurred for longer $T_{\mathrm{stim}}$ values, which are close to the $T_{\min }$.

We therefore conclude that the INa block increases LVC efficiency. We also find that in addition to the CB instability considered in Fig. 2, we also have a core-type instability that can be seen at longer periods.

#### Effects of amiodarone, verapamil and ICaL block

 Here, we study how LVC is affected by verapamil, the block of ICaL and amiodarone in high and low concentrations. We link the effect of verapamil not to its specific concentration but to the block of two currents, ICaL and IKr. The results are summarised in Table 4 and Fig. 5. Figure 5 shows that unlike the block of INa, the interval of effective stimulation periods (IESP) narrowed when verapamil or a CaL-blocker was applied.

**Table 4:**
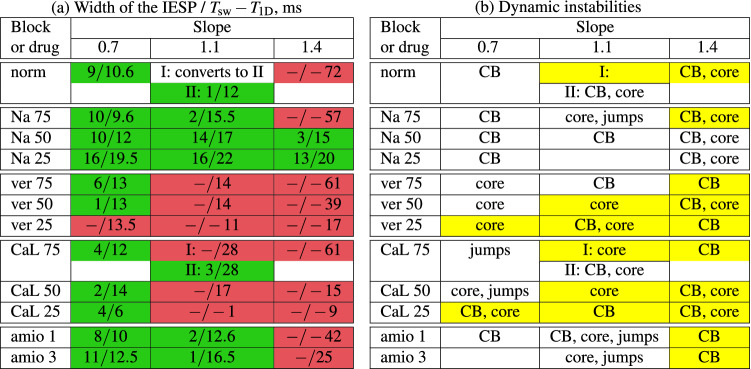
Results of the LVC.

Green, success; red, failure due to break up; yellow, meandering. Distinct spiral-wave solutions within a certain model are marked by I and II. Spiral wave period was averaged in cases of meandering. All boundaries of successful LVC periods were determined with an accuracy of 0.5 ms. The typical number of probed initial pacing periods was about 10–15.

The detailed results summarised in Table 4 are coded in the following way. Subtable 4a has green cells for cases when LVC was successful for at least one pacing period. The red colour marks the cases when LVC was not possible for all periods we tried. We also show two numbers, the first of which indicates the width of the period intervals of succesfull LVC (IESP; in red cells, IESP is absent so we write − there). The second number indicates $T_{\mathrm{sw}}-T_{\mathrm{1D}}$ for the given parameter set. Note that the second number is negative when $T_{\mathrm{sw}}< T_{\mathrm{1D}}$.

Observed instabilities are marked as ‘core’ or ‘CB’ in subtable 4b. Yellow cells indicate meandering of the spiral wave. For example, the cells of slope 0.7 and verapamil 75 are green with instability ‘core’ and two numbers 6 and 13. This means that the LVC was successful, the width of the interval was 6 ms and $T_{\mathrm{sw}}- T_{\mathrm{1D}}=13\,\mathrm{ms}$. The case CaL75 with slope 1.1 has two spiral wave solutions, I with meandering and II, a stable spiral without meander. Protocol S1S2 resulted in Solution I. Periodic pacing led to the occurrence of two new spiral waves, one of which annihilated with the original spiral wave, and the other had a circular tip trajectory and thus presented Solution II.

Now let us analyse the data presented in Table 4. For slope 0.7 we see, as discussed above, that the INa block increased the LVC efficiency. Amiodarone increased both IESP and $T_{\mathrm{sw}}- T_{\mathrm{1D}}$. The effects of the ICaL block and verapamil were not so straightforward. We see that all three examined values of the ICaL block caused a decrease in the LVC efficiency; however $T_{\mathrm{sw}}- T_{\mathrm{1D}}$ increased in cases CaL75 and CaL50 and decreased in case CaL25. Verapamil decreased the interval of successful LVC (IESP) but slightly increased $T_{\mathrm{sw}}- T_{\mathrm{1D}}$. We observed instabilities of the core type.

For slope 1.4, the effect of the INa block was discussed above. The ICaL block, verapamil and amiodarone had a stabilizing action, as they reduce the gap between $T_{\mathrm{sw}}$ and $T_{\mathrm{1D}}$. Unfortunately, such reduction was not sufficient for successful LVC.

For slope 1.1, the effects were basically similar to those for slope 0.7.

Table 4b shows observed dynamic instabilities. We clearly see that the INa block and amiodarone prevented the meandering and verapamil and ICaL block provoked it. We see a tendency that the instability for lower slopes was mainly of one type, and for higher slopes two types were observed depending on the pacing period. If we saw instabilities of several types, usually smaller pacing periods led to conduction blocks, longer periods led to core types, and even longer periods led to jumps.

### Induced drift speed analysis

**Figure 8:**
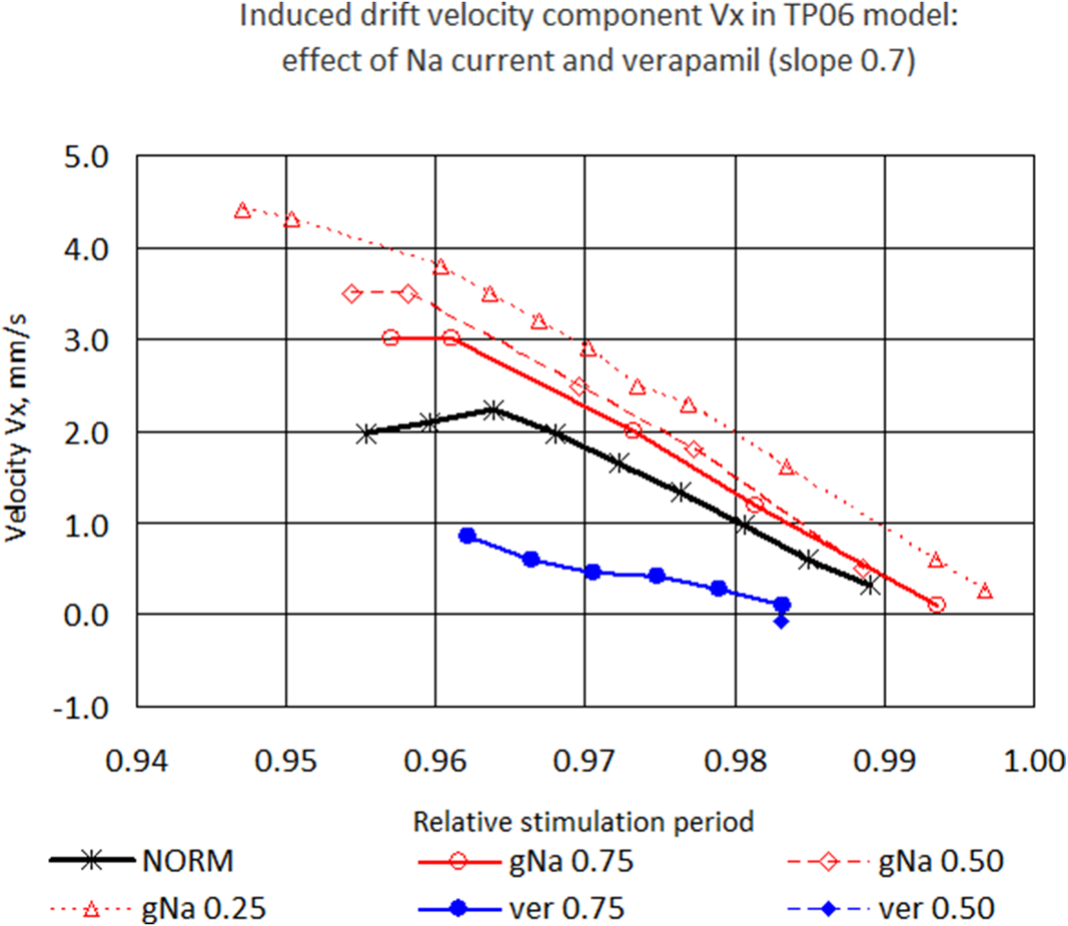
Longitudinal velocity component of the induced drift. Slope 0.7.

**Figure 9:**
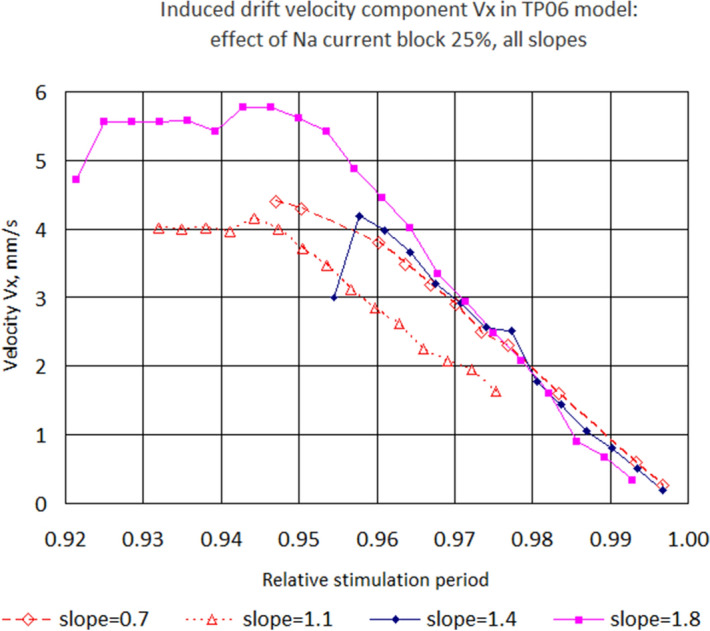
Drift velocity component *Vx* for Na25 and four different slopes.

For the parameter values where the IESP was sufficiently large, we plotted the dependency of the induced drift velocity component $V_x$ on the stimulation period. Figure 8 shows $V_x$ for slope 0.7. We see that the INa block made the IESP wider and accelerated the induced drift, and verapamil had the opposite effect. Figure 9 shows velocity $V_x$ for slopes from 0.7 to 1.8 for Na25. We see that the slope did not affect the velocity much. Therefore, the main parameter that affected the drift velocity was the stimulation period.

## Discussion and conclusions

### LVC and dynamic instabilities

In this paper, we performed a detailed study of LVC via high-frequency pacing in a 2D TP06 model of human cardiac tissue. We found that LVC can eliminate a spiral wave by pushing it towards the boundary of the tissue where the spiral wave disappears. We found that the main condition for successful elimination is possibility to force cardiac tissue with a faster period than the period of the spiral wave. However, LVC can also fail for several reasons, the main one being the onset of various instabilities. The most studied dynamic instability in cardiac tissue is associated with alternans, which occurs at a steep slope of the APD restitution curve^34,35^. Therefore, we performed studies of three sets of model parameters that have different slopes of the APD restitution curve, 0.7, 1.1 and 1.4. We found that an increase in the slope from 0.7 to 1.1 substantially reduced the efficiency of LVC and that LVC was not possible for the slope 1.4. To characterize the observed effects quantitatively, we used two parameters; one was the period of spiral wave $T_{\mathrm{sw}}$ and the other was the minimal period at which cardiac tissue could be stimulated $T_{\mathrm{1D}}$. In the absence of dynamic instabilities, $T_{\mathrm{1D}}$ equals the refractory period of cardiac tissue defined as the endpoint of the dynamic APD restitution curve. However, $T_{\mathrm{1D}}$ could be substantially longer in the presence of instabilities. We show that the necessary condition for successful LVC is a gap between the periods, $T_{\mathrm{sw}}>T_{\mathrm{1D}}$. This criterion explains the failure of LVC at the restitution curve slope of 1.4, as here $T_{\mathrm{sw}}<T_{\mathrm{1D}}$. However, further study showed that the difference between $T_{\mathrm{sw}}$ and $T_{\mathrm{1D}}$ is not the only parameter that determines the success of LVC. We found that the interval of successful LVC periods can be substantially shorter due to another type of 2D instability. In particular, we found that although the plane waves propagated stationarily and stably without an existing spiral wave, the presence of the spiral wave caused instabilities close to the spiral wave core (core-type instabilities) and that this made LVC impossible. The presence of this instability could substantially reduce the window of successful LVC periods; for example, the baseline model SL11 had $T_{\mathrm{sw}}$ longer than $T_{\mathrm{1D}}$ by 12 ms, while the successful LVC interval was only 1 ms.

Note that the conduction block instability, in particular the onset of new wavebreaks, occurs as a result of stimulation at the vulnerable phase, which has been known in electrophysiology since 1939^36^ and was directly demonstrated for high-frequency pacing in LR1 model in^37^.

### Effect of the stimulation protocol

Successful LVC relies on at least two important processes. First, it is necessary to stimulate the cardiac tissue with a period shorter than the period of the arrhythmia. Second, such a fast wavetrain must eliminate the arrhythmia source. Our study mainly focused on the second part, to investigate the interaction of a spiral wave with higher frequency waves. To do this, we used a special protocol to generate high-frequency waves. Special initial conditions provided that the wavetrain captured an area around the electrode, as described in the Methods section. We understand that this procedure is difficult to realise practically, but we wanted to decouple the problem of creation of high frequency from the problem of interaction of such a wavetrain with a spiral wave. However, we also performed simulations of LVC for more realistic pacing procedure so the tissue with a spiral wave was paced with the constant period Tstim (i.e. without that special procedure described above). We did it for SL07 and two periods: 228 and 235 ms. Total stimulation duration was 30 s. For each period, we performed 100 simulations with different initial conditions. For a period of 228 ms, we observed 77% successful elimination of the spiral; however, for the period of 235 ms, it occurred only in 27% of cases. Although these numbers do not look high, especially for the period of 235 ms, we found that the main issue here was the duration of the stimulation. If we continued stimulation for 120 s in both cases, we observed 100% efficiency. The observed difference between the results for two periods for 30-s stimulation was that the waves from the electrode reached the spiral wave core faster, and the induced drift had a higher velocity for the period of 228 ms than 235 ms. Note, however, that immediate pacing with a short constant period is not the best strategy and can result in the Wenckebach effect due to APD restitution, as described in^38^, and LVC will not be possible for that stimulation protocol. Thus, the stimulation protocol is an important factor for the efficiency of LVC, and it should be addressed in a subsequent study.

Note that sometimes LVC can be successful for any stimulation period due to different mechanisms. We saw such situations in the previous studies of LVC in the TP06 model. For example, we reported accidental wave annihilations for relative pacing periods from 192 ms to 245 ms^25^. Because stimulation periods below 227 ms display the Wenckebach effect, the effective periods of stimulation in many cases were above 384 ms and, thus, longer than the period of arrhythmia. However, LVC can still be successful here due to a completely different mechanism (spontaneous annihilation). It will be interesting to study if such indirect mechanisms can play an important role in the success of LVC.

### Effect of channel blockers and drugs

We studied how the block of INa and ICaL and the application of amiodarone and verapamil affect LVC efficiency. We found that the block of INa was always beneficial because it either increased the window of successful LVC periods or made LVC possible for the parameter set SL14. In all cases, the effect was achieved by increasing the difference between $T_{\mathrm{sw}}$ and $T_{\mathrm{1D}}$. For example, for the slope 1.4, the $T_{\mathrm{sw}}$ and $T_{\mathrm{1D}}$ difference changed from $-72$ ms in normal conditions to +15 ms for Na50. We also observed a positive effect of amiodarone. The effects of the ICaL block and verapamil were more complex. It seems that these actions somehow increased instabilities in cardiac tissue and made LVC less efficient in most cases. However, most of these instabilities were of the core type, and the difference between $T_{\mathrm{sw}}$ and $T_{\mathrm{1D}}$ could even increase with the drug application. The mechanisms of such effects are not completely clear. It is generally considered that the block of ICaL makes the APD restitution curve more flat and reduces dynamic instabilities. Stamp et al.^12^ claim that it can even increase the efficiency of LVC in an LR-I model. Note, however, that ‘core’-type instabilities were not observed in^12^, which may be related to the simplified description of ICaL in the LR-I model. It would be interesting to study the effects of an ICaL block and verapamil on LVC in other models of human cardiac tissue with complex intracellular Ca dynamics, such as the Grandi model^39^ and the ORd model^40^.

### Outlook and Limitations

We found that LVC in human cardiac tissue may effectively remove spiral waves. However, the window of successful LVC periods was quite short, only about 5–10 ms in most cases. Drugs such as INa blockers and amiodarone could increase the efficiency of LVC. Therefore, we suggest that the combination of LVC with drugs, particularly class I anti-arrhythmics^41^, can increase LVC efficiency. However, further research, both theoretical and experimental, should be undertaken.

Although in our paper we performed studies of LVC with a wide range of parameters, several other factors should be considered. We did not consider cases with a spontaneous break-up of the spiral wave. Such a break-up in the TP06 model was observed when the slope was 1.8. We performed pilot studies for the APD restitution curve slope of 1.8; however, LVC was always ineffective if ionic currents were unchanged. Therefore, further studies are necessary to determine if high-frequency pacing can be used in such conditions. We considered homogeneous cardiac tissue without anisotropy. Each of the factors of ionic heterogeneity^42,43^, non-conductive obstacles^44,45^, anisotropy^46,47^ and 3D effects^48^ can result in the onset of a new spiral wave under high-frequency pacing, which can interfere with the LVC process and make it more difficult.

A large class of cardiac arrhythmias are associated with anchored spiral waves and anatomical re-entry. Here, LVC should first detach the rotating wave from the obstacle and only after that can the spiral wave be successfully removed^14,49,50^. However, such studies have not yet been performed for human cardiac tissue models. It would therefore be interesting to perform such research.

In this study, we used only a linear electrode to remove spiral waves, as in the original paper^10^. Similar studies for point electrodes and other electrode shapes should also be performed to find how the number, location and shape of electrodes can affect LVC efficiency.

In this study, we focused on one possible mechanism of cardiac arrhythmias which occur due to spiral waves (also known as rotors or functional re-entry). We mainly considered the case of a single rotating spiral wave. Note that many cardiac arrhythmias occur due to different mechanisms, e.g. as a result of focal activity^51^ or rotational activity different from spiral waves, such as anatomical re-entry^52^. Many arrhythmias, such as VF and AF, can also be organised by multiple sources^53,54^. Although the main aim of our research was a study of arrhythmia due to single spiral waves, we realise that not considering arrhythmia which occur due to different mechanisms is a substantial limitation of our study. Note, however, that although ODP can be applied to focal arrhythmias, the possible mechanisms of its action are likely to be at the single-cell depolarisation-repolarisation dynamics level and not in the processes of wave interaction at the tissue level which we addressed in our study. Thus, a study targeting focal sources must be designed and performed as separate research. A large class of cardiac arrhythmias are associated with anchored spiral waves and anatomical re-entry^52^. Here, LVC should first detach the rotating wave from the obstacle and only after that can the spiral wave be removed^44^. However, such studies have not been performed using human cardiac tissue models. It would be interesting to perform such research. Finally, the application of ODP to VF and especially AF is an extremely interesting and important avenue of research. We partially addressed this issue in our research by considering a model of type SL14, which produces excitation patterns similar to that of cardiac fibrillation^28^. Unfortunately, LVC was never successful for those parameter values, and further research in that direction is needed.

Ultimately, we used a monodomain description for cardiac tissue. Most studies on defibrillation have used a bi-domain description; high-voltage shocks produce virtual electrode patterns in the heart. Although we did not use high voltages to initiate the wave, it would still be interesting to perform studies using a bi-domain model to see if it can result in new types of dynamics.

## Supplementary information

Supplementary tables

Supplementary video 1

Supplementary video 2

Supplementary video 3

Supplementary video 4
