# Supplementary material for: Overdrive pacing of spiral waves in a model of human ventricular tissue
Source: Sci Rep. 2020 Nov 26;10:20632. doi: 10.1038/s41598-020-77314-5 (PMC7691998; doi:10.1038/s41598-020-77314-5)
Supplement: Supplementary file 1 — Supplementary tables [file 41598_2020_77314_MOESM1_ESM.pdf]

Overdrive pacing of spiral waves in a model of human ventricular tissue: Supplementary information

Sergei F. Pravdin, Timofei I. Epanchintsev, Alexander V. Panfilov

A Pacing parameters

Supplementary Table S 1. Pacing parameters for the SL07 models

TP06 modification	Stimulation current I_{st}, μA/cm^2	S2 zone α_{S2}	S2 moment t_{S2}, ms	Pacing start τ_0, ms	Initial pacing period, ms	Spiral wave period T_{sw}, ms
norm	50	0.5	340	2106	234	237.6
Na75	50	0.5	347	1185	237	246.6
Na50	50	0.5	360	2000	252	264
Na25	50	0.5	360	2000	290	306.5
ver75	50	0.7	350	2000	248	238
ver50	35	0.7	368	4240	235	237
ver25	50	0.7	391	2000	283	243.5
CaL75	30	0.7	335	2000	218	225
CaL50	30	0.6	335	5500	212	215
CaL25	30	0.7	300	2000	198	205
am1	50	0.6	340	2000	242.5	250
am3	50	0.6	340	2000	253	257.5

Supplementary Table S 2. Pacing parameters for the SL11 models

TP06 modification	Stimulation current, μA/cm^2	S2 zone α_{S2}	S2 moment t_{S2}, ms	Pacing start τ_0, ms	Initial pacing period, ms	Spiral wave period T_{sw}, ms
norm	35	0.7	377	2000	230	I: 260; II: 240
Na75	40	0.6	370	2000	254	253.5
Na50	40	0.6	380	2000	273	273
Na25	40	0.6	410	2000	323	323
ver75	35	0.6	400	11000	230	239
ver50	40	0.6	380	4000	245	245
ver25	30	0.6	2355	500	250 from $t = 500$ ms to $t = 2355$ ms; 233 from $t = 4870$ ms	247
CaL75	30	0.6	365	2000	223	I: 240; II: 225
CaL50	40	0.6	320	4000	221	218
CaL25	30	0.6	350	4000	202	207
am1	30	0.6	380	3000	252	253.6
am3	40	0.6	360	4000	263	262.5

Supplementary Table S 3. Pacing parameters for the SL14 models

TP06 modification	Stimulation current, μA/cm^2	S2 zone α_{S2}	S2 moment t_{S2}, ms	Pacing start τ_0, ms	Initial pacing period, ms	Spiral wave period T_{sw}, ms
norm	35	0.6	377	3070	222	243
Na75	35	0.5	390	4000	240	250
Na50	40	0.5	402	5000	257	256
Na25	35	0.7	432	2250	309	308
ver75	35	0.6	377	3330	228	231
ver50	35	0.6	377	2100	230	237
ver25	35	0.6	377	2000	236	241
CaL75	30	0.6	377	2490	223	215
CaL50	35	0.6	377	2000	196	210
CaL25	35	0.6	310	1180	200	196
am1	40	0.6	390	5000	240	255
am3	40	0.6	380	5000	240	250

B Role of the pacing start, period and spiral wave position: statistics

Here we show results for a straightforward stimulation with a constant period. We made two series of simulations with a TP06 model and isotropic medium. The first series used a stimulation period of 228 ms, which is about 0.96 of the spiral wave period. The second series used a stimulation period of 235 ms or about 0.99 of the spiral wave period. We varied the start of the pacing from 1900 ms to 2125 ms with a step of 25 ms and time of S2 stimulus (which changed the spiral wave position) from 315 ms to 360 ms with a step of 5 ms in both series. Totally, each series consisted of 100 simulations. The pacing was given from 2 s to 30 s and the result was assessed at 31 s, that is, after 1 s of the silence of the electrode. Our statistics are shown in Supplementary Table S 4. Note that the row 'Spiral removed' shows results for spiral elimination due to both superseding by overdrive pacing and annihilation with other spirals. The outcome 'Still a single spiral' in many cases just indicates that 30 s was not sufficient for spiral removal, thus we have much more such cases for the stimulation period of 235 ms when the induced drift speed was much lower. New spirals occurred here at the electrode as a result of simulation at the vulnerable phase. The results for stimulation with a constant period are provided here just as a benchmark, as such straightforward stimulation protocol is used neither in clinical nor in most numerical settings.

Supplementary Table S 4. Statistics of pacing with different spiral wave positions and pacing starts (SL07, norm)

Result (# of cases and percentage)	Period	
	228 ms	235 ms
Spiral removed	77	27
Still a single spiral wave	19	56
One new spiral wave	4	15
Two new spiral waves	0	2

The pacing with a period of 228 ms was successful only in a few cases because the long electrode caused new spiral waves to appear via a mechanism similar to the one used in the S1S2 protocol. When the pacing phase was appropriate, new spiral waves did not emerge, and we saw the growth of the electrode-controlled area in the domain toward the spiral wave core and then the induced drift. After the original spiral wave was superseded, the pacing of the 'empty' domain caused two new spiral waves to appear; therefore, a timely stop of the pacing is also important, at least in our experiments.

The stimulation with a period of 235 ms removed the spiral wave only accidentally when it annihilated with a new spiral wave generated at the electrode. The complete failure of LVC with that period was due to the very slow induced drift of the original spiral. In a few cases of the 56 cases, when still a single spiral wave persisted, a pacing duration of up to 1 min could move the spiral wave to the domain boundary.

In addition, the pacing with a constant period was ineffective in many cases with changed slopes and ionic currents due to the unreadiness of the cells to be stimulated faster than the spiral wave period. Therefore, pacing with a period essentially lower than T_{sw} was done using a slow decreasing of the period.

C Phase singularities in the SL14 model

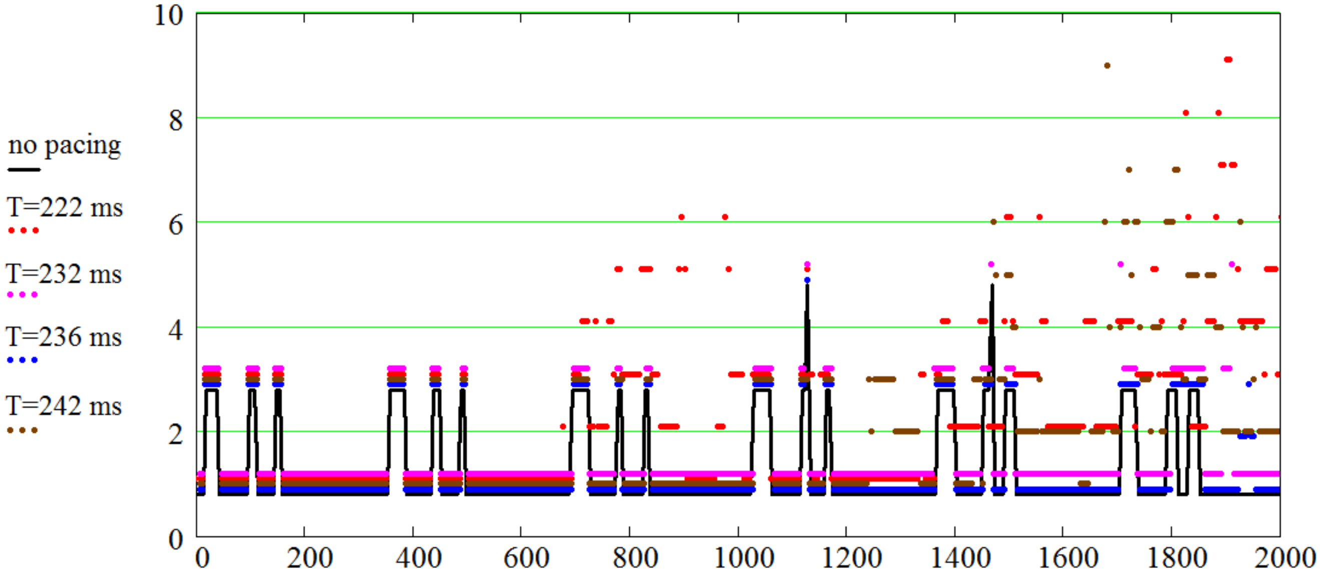

Supplementary Figure S 1. Number of phase singularities found against time for a spiral wave in the SL14 model without pacing (black line) and with (colourful points; T in the legend is pacing period). The black, blue, brown, red and magenta points are shifted by -0.2, -0.1, 0, $+0.1$, $+0.2$ to make them more clear. The X-axis shows time (ms). The pacing started at 0 ms.

D Animation files

1. Example of successful LVC with a period shorter than that of the spiral wave. SL07, norm, $T_{\text{stim}} = 228$ ms.
2. Example of unsuccessful LVC. SL14, norm, $T_{\text{stim}} = 232$ ms.
3. Dynamic instability of the core type. SL11, Na75, $T_{\text{stim}} = 239$ ms.
4. Example of transient core-type dynamic instability (jumps-type). The spiral wave breaks up near its core as a result of the stimulation. The core then moves to another position and the wavefront annihilation zone shifts back toward the electrode. SL11, Na75, $T_{\text{stim}} = 247$ ms.
